# Binding of ApoE Isoforms to Aβ Peptides and Effects on Their Fibrillization

**DOI:** 10.1021/acsomega.5c12353

**Published:** 2026-02-03

**Authors:** Merlin Sardis, Andra Noormägi, Jüri Jarvet, Astrid Gräslund, Sebastian K. T. S. Wärmländer, Vello Tõugu, Peep Palumaa

**Affiliations:** † Department of Chemistry and Biotechnology, 54561 Tallinn University of Technology, Tallinn 19086, Estonia; ‡ Department of Biochemistry and Biophysics, 7675 Stockholm University, Stockholm 106 91, Sweden; § The National Institute of Chemical Physics and Biophysics, Tallinn 12618, Estonia

## Abstract

Alzheimer’s disease (AD) is the most widespread neurodegenerative disease, strongly linked to amyloid depositions in the brain consisting of amyloid β (Aβ) peptides. The likelihood of developing late-onset Alzheimer’s disease (LOAD) is influenced by the specific isoforms of apolipoprotein E (ApoE), with ApoE4 being the strongest known genetic risk factor for LOAD. Strong evidence suggests that ApoE impacts AD by modulating Aβ aggregation and clearance, although the precise molecular mechanisms remain incompletely understood. Microscale thermophoresis (MST) is a powerful technique for characterizing molecular interactions in solution, which has been used to determine various binding constants, although not the binding of ApoE to Aβ peptides. MST results show that ApoE isoforms bind Aβ1–40 and Aβ1–42 with low micromolar affinity. For Aβ1–42, ApoE3 shows the strongest binding (*K*_d_ = 0.72 μM) and ApoE4 shows the weakest binding (*K*_d_ = 2.80 μM). For Aβ1–40, ApoE4 shows the strongest binding (*K*_d_ = 1.59 μM) and ApoE2 shows the weakest binding (*K*_d_ = 5.29 μM). The MST results show that ApoE interacts with Aβ peptides at supraphysiological peptide concentrations. However, ApoE inhibited the fibrillization of Aβ1–42 peptide at substoichiometric concentrations, which might be related to blocking Aβ fibril elongation in vivo. The estimated IC_50_ values indicate that ApoE4 has a slightly stronger, and ApoE2 a slightly weaker, inhibitory effect on Aβ1–42 fibrillization.

## Graphical abstract

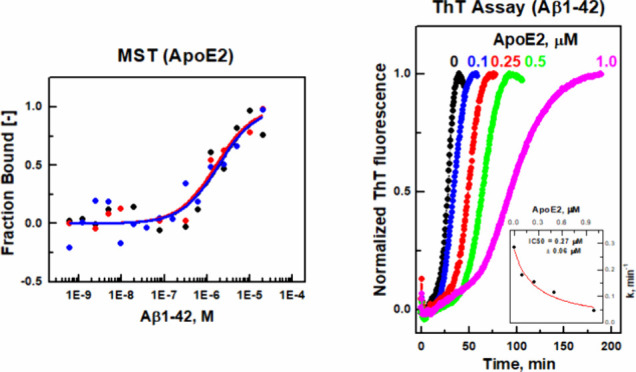

## Introduction

Alzheimer’s disease (AD) is the most prevalent neurodegenerative disorder, characterized by the occurrence of amyloid deposits in the brain composed of Aβ (amyloid-beta) peptides. The risk of developing late-onset Alzheimer’s disease (LOAD) is dependent on the isoforms of apolipoprotein E (ApoE) that individuals inherit. ApoE exists primarily in three isoforms: ApoE2 (Cys112, Cys158), ApoE3 (Cys112, Arg158), and ApoE4 (Arg112, Arg158).^1^ ApoE is colocalized with Aβ in senile plaques of AD and has been shown to bind tightly to immobilized Aβ peptide.^2^ Notably, the presence of ApoE4 is recognized as the highest genetic risk factor for developing LOAD.^2^

There is substantial evidence that ApoE influences AD by affecting Aβ aggregation and clearance;^3^ however, the molecular mechanism underlying this remains poorly understood. The direct interaction of ApoE with Aβ peptides has been studied mostly by ELISA; however, a few reports have used dual polarization interferometry (DPI)^4^ and surface plasmon resonance.^5^ According to ELISA, the dissociation constant values for ApoE-Aβ complexes are 48 nM (ApoE2), 63.7 nM (ApoE3), and 75.9 nM (ApoE4).^6^ A similar study using cell-derived ApoE (Sf9, HEK, and RAW cells) reported the following values: 13.3 nM (ApoE3) and 13.9 nM (ApoE4) for Aβ1–42 and 9.3 nM (ApoE3) and 10.3 nM (ApoE4) for Aβ1–40.^7^ According to Sadowski et al., the dissociation constant of the Aβ1–40 complex with lipidated ApoE4 determined by ELISA is 14.7 nM.^8^ One study using DPI determined the *K*_d_ values for ApoE binding to Aβ1–40 to be 251 nM (ApoE2), 40 nM (ApoE3), and 24.6 nM (ApoE4).^4^ Surface plasmon resonance was used by Rahman et al. to determine a 3 nm *K*_d_ value for ApoE binding to Aβ1–42 protofibrils.^5^ Liu et al. used ELISA to map the binding regions between ApoE and Aβ, identifying residues 244–272 on ApoE and 12–28 on the Aβ peptide as the interacting sites.^9^ Using a different approach, MD simulations, it was proposed that Aβ residues Asp1 and Asp23 might interact with cationic residues of ApoE, and thereby perturb its salt bridge network, especially in ApoE4.^10^

The effect of ApoE on Aβ fibrillization has been widely studied; however, the results are conflicting. Some authors have reported that ApoE has a direct role in promoting and accelerating fibril formation of Aβ1–40, where ApoE4 was more efficient than ApoE3 at enhancing amyloid formation.^11^ Sadowski et al. also showed that adding lipidated human ApoE4 significantly increased the amount of Aβ1–40 fibrils.^8^ Lipidated ApoE has been shown to increase Aβ oligomer levels in an isoform-dependent manner, with lipidated ApoE4 exerting the greatest effect on Aβ oligomer formation.^12^ Liao et al. claimed that ApoE-mediated plaque formation may be the result of ApoE aggregation, as evidenced by their observation that antihuman ApoE4 antibody binds nonlipidated and aggregated ApoE4 from amyloid plaques in mice, reducing Aβ deposition.^13^

On the contrary, Evans et al. showed that ApoE inhibits amyloid formation at substoichiometric levels.^14^ Furthermore, Garai et al. reported that at low concentrations, ApoE binds to and stabilizes Aβ oligomers, whereas at higher concentrations, it interacts with Aβ fibrils, stabilizing them and thereby inhibiting further fibril formation.^15^ In addition, ApoE has been shown to slow down the oligomerization of Aβ1–40 in solution.^16^ The findings of Ghosh et al. also support the inhibitory effect, as they show that Aβ1–42 aggregation is delayed dramatically in the presence of stoichiometric concentrations of both lipid-free and lipidated ApoE, and that ApoE preferentially interacts with fibrillization intermediates rather than with Aβ monomers.^17^ Recently, the same group reported that lipidated ApoE inhibits the elongation of Aβ1–42 fibrils in an isoform-dependent manner, where ApoE2 and ApoE3 exhibited the strongest inhibitory effects, while secondary nucleation was largely unaffected.^18^ According to Islam et al., ApoE inhibits the process of fibril elongation and prevents amyloid maturation.^19^ Xia et al. indicated that all ApoE isoforms associate with Aβ in the early stages of fibrillization and then fall away as fibrillization occurs.^3^ It has also been shown that ApoE modulates the aggregation, clearance, and toxicity of Aβ in an isoform- and lipidation-specific way, by selectively removing nonlipidated ApoE4-Aβ coaggregates and enhancing the clearance of toxic Aβ by glial cells.^3^ Ghosh et al. found that both ApoE3 and ApoE4 suppress, at substoichiometric levels and in a concentration-dependent manner, also the aggregation of other proteins, such as the AD-related proinflammatory protein S100A9.^20^

Microscale thermophoresis (MST) is a powerful method for characterizing molecular interactions in solutions.^21^ To date, MST has been used to study ApoE binding to complement regulator factor H,^22^ various chemical probes,^23^ and α-synuclein.^24^ In this study, we utilized MST to examine the interaction between Aβ peptides and ApoE and determined the effect of ApoE proteins on the fibrillization of Aβ1–42 using the ThT assay. Our results indicate that Aβ peptides interact with ApoE at micromolar concentrations, whereas ApoE inhibits fibrillization of Aβ1–42 at substoichiometric concentrations.

## Results and Discussion

### Microscale Thermophoresis

The binding of Aβ peptides to ApoE isoforms was studied using MST with fluorescently labeled ApoE (20 nM) isoforms (E2/E3/E4) as the target and Aβ1–42 or Aβ1–40 as the ligand.

The obtained *K*_d_ values remained in the range of 1–2 μM (Figure 1, Tables 1 and 2) except that for ApoE2 binding to Aβ1–40 (Figure 1d, Table 2), characterized by the largest *K*_d_ value, approximately 5 μM. The smallest *K*_d_ value was below 1 μM and was observed for ApoE3 binding to Aβ1–42.

**Figure 1:**
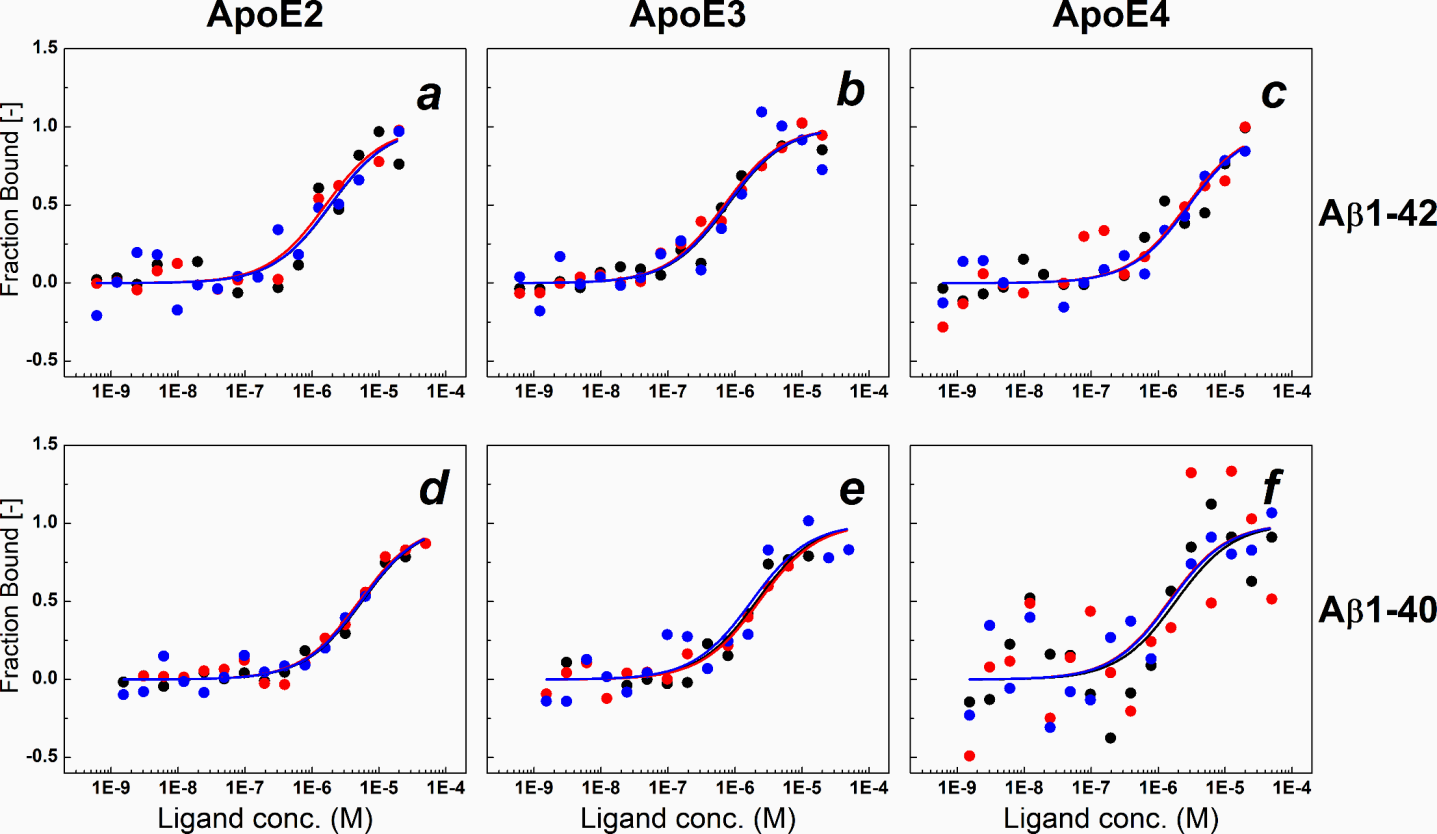
Binding of Aβ1–42 (a–c) and Aβ1–40 (d–f) to ApoE2 (a, d), ApoE3 (b, e), and ApoE4 (c, f). The experimental MST data from three repeats are represented as points in black solid circle, red solid circle, and dark-blue solid circle. Lines correspond to the fitted results. Conditions: 20 nM labeled ApoE2/E3/E4, Aβ1–40 (0.003–50 μM), and Aβ1–42 (0.0012–20 μM) in 50 mM HEPES, 150 mM NaCl, pH = 7.4, containing 10% glycerol and 0.1% Pluronic F-127, 23 °C.

**Table 1:** *K*_d_ and RMSE Values for Binding of ApoE Isoforms to Aβ1–42 (Figure 1a–c)

**# (Aβ1–42)**	**ApoE2 *K***_**d**_ **(μM) ± RMSE**	**ApoE3 *K***_**d**_ **(μM) ± RMSE**	**ApoE4 *K***_**d**_ **(μM) ± RMSE**
I	1.85 ± 0.13	0.76 ± 0.07	2.91 ± 0.10
II	1.87 ± 0.13	0.73 ± 0.16	2.70 ± 0.16
III	1.58 ± 0.09	0.68 ± 0.06	2.77 ± 0.11

**Table 2:** *K*_d_ and RMSE Values for Binding of ApoE Isoforms to Aβ1–40 (Figure 1d–f)

**# (Aβ1–40)**	**ApoE2 *K***_**d**_ **(μM) ± RMSE**	**ApoE3 *K***_**d**_ **(μM) ± RMSE**	**ApoE4 *K***_**d**_ **(μM) ± RMSE**
I	5.52 ± 0.04	2.07 ± 0.10	1.82 ± 0.28
II	5.02 ± 0.06	2.27 ± 0.07	1.44 ± 0.38
III	5.34 ± 0.08	1.74 ± 0.16	1.50 ± 0.23

Our results show that the interaction of monomeric Aβ and ApoE, characterized by *K*_d_ values determined by MST experiments, shows significantly weaker affinity than that estimated earlier by ELISA and DPI. The observed difference can be attributed to the nature of the measurements: MST measurements determine interactions in solutions with monomeric Aβ, whereas ELISA and DPI evaluate the affinity of ApoE for the peptide immobilized on a solid surface. It is well-known that affinity values vary depending on the experimental conditions, molecular form of the peptide, and the technique used for the measurements.^17,25^ The current MST results indicate that the interaction of ApoE with the Aβ peptide occurs at supraphysiological micromolar concentrations, suggesting that the ApoE interaction with Aβ peptides is unlikely to occur under physiological conditions. However, such interactions might occur within the limited volume of synaptic clefts, where the Aβ concentration temporarily can reach micromolar concentrations.^26^ Measured *K*_d_ values varied only slightly, with differences ranging from 3-fold to 4-fold, depending on the type of ApoE or Aβ isoform. ApoE4 exhibits the weakest binding to Aβ1–42 but the strongest binding to Aβ1–40. The experiments were conducted using lipid-free ApoE, which can bind nonpolar substrates, and it could be suggested that in the case of lipidated ApoE, the *K*_d_ values may be even higher. Our findings align with those of Verghese et al., who used multiple biochemical and analytical techniques to demonstrate minimal binding of lipidated ApoE to Aβ peptide in physiological fluids, with a slight increase in binding observed when the lipidation level was reduced.^27^

### Fluorescence Spectrophotometry

The effect of ApoE isoforms on Aβ fibrillization was studied with fluorescence spectrophotometry using the ThT assay. The fibrillization of 5 μM Aβ1–42 was assessed in the absence and the presence of ApoE at concentrations ranging from 50 nM to 1 μM. The results indicate that the addition of as little as 100 nM ApoE2/E3/E4 (Figure 2a–c, dark-blue solid circle) to 5 μM Aβ1–42 inhibits Aβ1–42 fibrillization compared to the control (Figure 2a–c, black solid circle). As the ApoE concentration increases, the rate of fibrillization progressively declines, which shows that the effect is concentration-dependent. IC_50_ values with standard errors were determined from scatter plots and are 0.27 ± 0.06, 0.16 ± 0.03, and 0.12 ± 0.06 μM for ApoE2, ApoE3, and ApoE4, respectively. This suggests that ApoE4 exhibits the strongest inhibitory effect on Aβ fibrillization, followed by ApoE3 and ApoE2.

**Figure 2:**
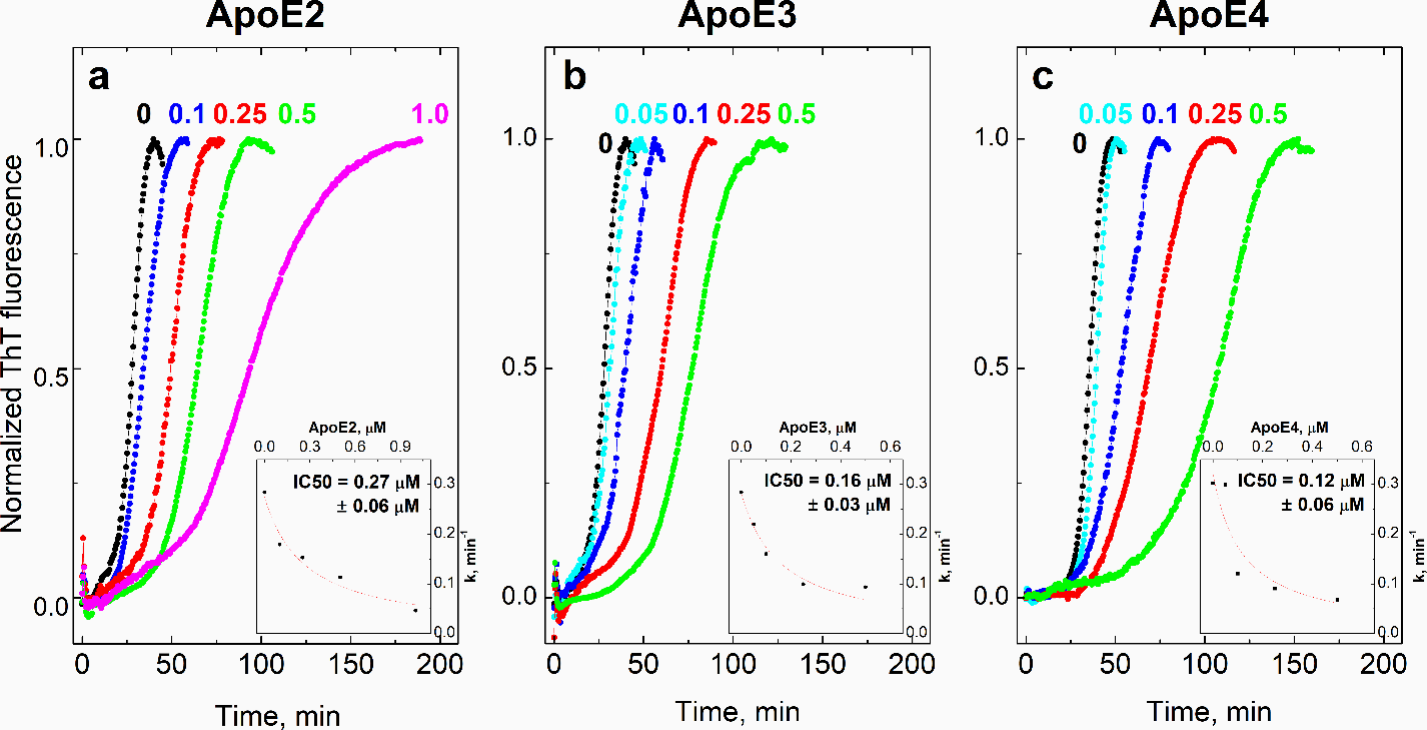
Fibrillization of 5 μM Aβ1–42 (control, black solid circle) in the presence of 0.05 μM (light-blue solid circle), 0.1 μM (dark-blue solid circle), 0.25 μM (red solid circle), 0.5 μM (green solid circle), and 1 μM (pink solid circle) ApoE2 (a), ApoE3 (b), and ApoE4 (c) in a normalized scale of fluorescence intensity (*F*/*F*_0_); *n* = 1 for all ApoE concentrations. Inset: Dependence of the fibrillization rate constant on the ApoE concentration. Conditions: 20 mM HEPES, 100 mM NaCl, pH = 7.4, 40 °C, constant stirring in the presence of 5 μM ThT.

The IC_50_ values for ApoE, determined from the inhibition of Aβ fibrillization, were lower than the *K*_d_ values. Notably, the inhibition occurred already at low substoichiometric concentrations: 50 times less ApoE (0.1 μM) reduces the rate of Aβ1–42 fibrillization by 1.5–2.5 times. The inhibitory effect of ApoE on the fibrillization rate of Aβ1–42 may be explained by ApoE blocking of the Aβ1–42 fibrillization sites. Recently, Dasadhikari et al. reported that lipidated ApoE inhibits Aβ1–42 fibril elongation, while secondary nucleation remains largely unaffected.^18^ Single fibril studies confirmed that inhibition of the elongation rate is proportional to the binding of ApoE to the terminal ends of the fibrils. The affinity constants of ApoE isoforms for fibril ends were isoform-specific, with ApoE4 exhibiting 4-fold weaker binding compared to ApoE2 and ApoE3.^18^ In our study, we observed rather similar inhibitory effects of different ApoE isoforms on the Aβ1–42 fibril elongation rate, falling within experimental uncertainty, whereas ApoE4 showed only slightly stronger inhibition. Fibrillization experiments, similar to MST experiments, were performed with lipid-free ApoE, and therefore, the results may be different in the case of lipidated ApoE forms.

The current results show that ApoE has a weak ability to interact with Aβ peptide monomers; however, its ability to interact with intermediates of Aβ fibrillization is substantially higher. Therefore, ApoE can block the fibrillization of Aβ peptides, where the effects of the different ApoE isoforms are quite similar. According to our results, the isoform-specific behavior of ApoE in the pathogenesis of AD is not linked to its interaction with Aβ monomers or aggregation intermediates. Still, it might be connected to other interactions.

The ApoE genotype may contribute to AD pathogenesis through several other distinct mechanisms. One hypothesis is related to the LDL receptor-related protein 1 (LRP1), which is involved in the internalization and degradation of Aβ ^28,29^ as well as in the uptake and spread of tau.^30,31^ ApoE has been shown to disrupt Aβ clearance from the brain,^32^ and blocking the ApoE/Aβ interactions resulted in enhanced Aβ clearance from the brain and decreased plaque deposition.^8,33^ Therefore, ApoE isoforms may influence Aβ metabolism by competing for the same clearance pathways in the brain.^27^ ApoE4-specific misfolded intermediate states shown by MD simulations can suppress the clearance of Aβ.^34,35^

Alternatively, there might occur ApoE isoform-specific microglial lipid droplet accumulation, which leads to tau phosphorylation and neurotoxicity in an ApoE-dependent manner.^36^ It is also known that ApoE4 increases tau pathogenesis and leads to increased astroglial- and microglial-mediated persistent inflammation, which leads to significant neurodegeneration in the presence of ApoE4.^37^ Yet, it remains a conundrum exactly how slight modifications in the protein sequence of ApoE isoforms have such a large impact on AD pathogenesis.

## Materials and Methods

Lyophilized Aβ1–40 and Aβ1–42 were purchased from r-Peptide (Watkinsville, GA, USA); ApoE isoforms (E2/E3/E4) were purchased from AlexoTech AB (Umeå, Sweden). The integrity of the proteins was verified by MALDI-MS.

1,1,1,3,3,3-Hexafluoroisopropanol (HFIP), HEPES, NaCl, NaOH, glycerol, NH_4_OH, and Thioflavin-T (ThT) were purchased from Sigma-Aldrich.

Pluronic F-127, labeling buffer (LB-NHS), RED-NHS second generation dye, B-column, and DMSO were included in Protein Labeling Kit RED-NHS second generation (MO-L011) and purchased from NanoTemper (NanoTemper Technologies, München, Germany).

### Sample Preparation

Lyophilized Aβ1–40 and Aβ1–42 were solubilized in HFIP at a concentration of 100 μM and divided into aliquots, and the HFIP was evaporated under vacuum. The tubes with the Aβ film were stored at −80 °C. HFIP treatment is used to disassemble peptide aggregates into monomers.

ApoE isoforms (E2/E3/E4) were dissolved in a 5 mM NaOH solution to a final concentration of 50 μM, divided into aliquots, and stored at −20 °C.

### Microscale Thermophoresis

ApoE isoforms were diluted to a final concentration of 10 μM with the labeling buffer (LB-NHS in MO-L011, NanoTemper) and labeled with the NHS reactive dye (30 μM, dissolved in DMSO) from Protein Labeling Kit RED-NHS second generation (MO-L011; NanoTemper, München, Germany). The labeling reaction was carried out for 30 min at 25 °C in the dark; the mixture was applied to the column according to the manufacturer’s instructions and eluted with 50 mM HEPES, 150 mM NaCl, pH 7.4, 10% glycerol, and 0.1% Pluronic F-127. 10% glycerol was added to the buffer to decrease the aggregation of ApoE. The labeling efficiency was estimated using a NanoDrop 2000c spectrophotometer (Thermo Scientific, Waltham, Massachusetts, USA) to be in the range of DOL = 0.5–1. Labeled ApoE isoforms were divided into aliquots and stored at −20 °C. Before use, the labeled ApoE isoforms were thawed, diluted to 40 nM in 100 mM HEPES, 300 mM NaCl, pH 7.4, 20% glycerol, and 0.2% Pluronic F-127 and centrifuged at 21,000 *g*, 4 °C, for 10 min.

HFIP-treated Aβ1–42 and Aβ1–40 aliquots were dissolved in 5 mM NaOH to final concentrations of 40 and 100 μM, respectively, incubated for 10 min on ice, and centrifuged for 10 min, 4 °C, 21,000 *g* before use. Since amyloid β tends to aggregate at neutral pH, the 16-point serial dilutions of Aβ1–40 (100 μM) and Aβ1–42 (40 μM) were made in 5 mM NaOH. Samples for measurement were prepared by mixing 10 μL of a 16-point serial dilution of Aβ1–40 (100 μM) or Aβ1–42 (40 μM) with an equal amount of labeled ApoE isoforms (40 nM) to the final concentration of Aβ1–40 and Aβ1–42 ranging from 50 μM to 3 nM and 20 μM to 1.3 nM, respectively. Samples were measured in 50 mM HEPES, 150 mM NaCl, pH 7.4, containing 10% glycerol and 0.1% Pluronic F-127. After mixing, the samples were instantly loaded into standard capillaries, inserted into the Monolith NT 115 system (NanoTemper Technologies, München, Germany), and measured using 20% LED and low MST intensity (20% IR-laser power), with temperature control at 23 °C. Results were analyzed with MO Affinity Analysis software and fitted by the *K*_d_ equation provided by the software. MST-on time used for analysis was 20 s. The *K*_d_ values, together with the root-mean-square error (RMSE), were estimated using MO Affinity Analysis software.

### Fluorescence Spectrophotometry

HFIP-treated Aβ1–42 was dissolved in 0.02% NH_4_OH, incubated for 10 min on ice, and diluted with 40 mM HEPES, 200 mM NaCl, pH = 7.4 to a final concentration of 5 μM Aβ1–42, 20 mM HEPES, 100 mM NaCl, pH = 7.4.

Fibrillization of Aβ1–42 was studied using the fluorescent ligand ThT, whose fluorescence intensity at 480 nm (excitation at 440 nm) is increased upon binding to amyloid fibrils. ThT fluorescence was monitored on a PerkinElmer (Waltham, MA, USA) LS55 fluorescence spectrophotometer in a 500 μL cuvette by constant stirring at 40 °C in the presence of 5 μM ThT. To study the effect of ApoE isoforms on Aβ1–42 fibrillization, Aβ1–42 fibrillization curves in the absence and presence of different concentrations of ApoE2, ApoE3, and ApoE4 were determined and fitted to the Boltzmann eq 1 by the program Origin 8.5 (OriginLab Corporation, USA):

$$y=\frac{A_{1}-A_{2}}{1+e^{(t-t_{0})\cdot k}}+A_{2}$$ 1

where *A*_1_ is the initial fluorescence intensity level, *A*_2_ corresponds to the fluorescence at maximal fibrillization level, *t*_0_ is the time *t* when fluorescence reached half-maximum, and *k* is the apparent rate constant for the growth of fibrils.

The half-maximal inhibitory concentration (IC_50_) values were calculated from the effects of ApoE isoforms on the apparent rate constant of fibril formation (IC_50k_) according to hyperbolic dose–response curves:

$$y=A_{2}-\frac{A_{2}\times c}{{\mathrm{IC}}_{50}+c}$$ 2

where *y* is the fluorescence intensity of ThT, *A*_2_ is the maximum value of ThT fluorescence, *c* is the concentration of the test substance, and IC_50_ is the concentration that reduces ThT fluorescence by 50%. Normalization of data and nonlinear regression analysis were carried out using the program Origin 8.5.
